# Management of penetrating cardiac injury and tricuspid regurgitation with extracorporeal-membrane oxygenation (ECMO): a case report

**DOI:** 10.1186/s13019-024-02557-6

**Published:** 2024-02-06

**Authors:** Alexandros N. Karavas, Keeyon Olia, Dane Scantling, Jacob Nudel, Jacob Kriegel, Niloo M. Edwards

**Affiliations:** 1 Division of Cardiac Surgery, Boston Medical Center, Boston University Chobanian and Avedisian School of Medicine, Boston, MA 02118 USA; 2 Department of Medicine, University of New England College of Osteopathic Medicine, Biddeford, ME 04005 USA; 3 Division of Trauma and Acute Care Surgery, Boston Medical Center, Boston University Chobanian and Avedisian School of Medicine, Boston, MA 02118 USA; 4 Department of Surgery, Boston Medical Center, Boston University Chobanian and Avedisian School of Medicine, Boston, MA 02118 USA

**Keywords:** Penetrating cardiac trauma, Ballistic injury, Extracorporeal membrane oxygenation, Tricuspid regurgitation, Traumatic valve injury

## Abstract

**Background:**

Gunshot wounds (GSW) to the heart are lethal, and most patients die before they arrive to the hospital. Survival decreases with number of cardiac chambers involved. We report a case of a 17-year-old male who survived a GSW injury involving two cardiac chambers with acute severe tricuspid regurgitation (TR) who subsequently developed cardiogenic shock requiring extracorporeal membrane oxygenation (ECMO) support.

**Case Presentation:**

A 17-year-old male sustained a single gunshot wound to the left chest, resulting in pericardial tamponade and right hemothorax. Emergency sternotomy revealed injury to the right ventricle and inferior cavoatrial junction with the adjacent pericardium contributing to a right hemothorax. The cardiac injuries were repaired primarily. Tricuspid regurgitation was confirmed immediately postoperatively. Five days after presentation, the patient developed cardiogenic shock secondary to TR requiring emergent stabilization with ECMO. He subsequently underwent successful tricuspid valve replacement.

**Conclusions:**

This is the first report to our knowledge of successful ECMO support of severe TR due to gunshot injury to the heart.

## Background

Firearm related deaths in the US have increased in frequency over the last decade and represent a significant public health threat [1–3]. Some reports reveal firearms as a leading cause of death among children [4]. Traumatic cardiac injuries are lethal with up to 90% of patients dying before they reach a hospital, and mortality is significantly increased when multiple cardiac chambers are involved [3, 5, 6]. Extracorporeal support has been used infrequently in the management of penetrating cardiac injuries; its limited use is likely due to injury acuity, resource availability, and logistical factors. However, with increased acceptance of low to no anticoagulation while on extracorporeal membrane oxygenation (ECMO), several reports have described its utility as an important additional resource for the management of trauma patients [7]. 

This is the first report to our knowledge of a ballistic injury to the heart resulting in cardiogenic shock due to severe tricuspid regurgitation (TR) that required veno-arterial (VA) ECMO support prior to its definitive management.

## Case presentation

A 17-year-old male presented as an emergent transfer from an outside hospital after having sustained a single gunshot wound to the left chest. He was endotracheally intubated at the outside hospital, massive transfusion protocol was initiated, and a right sided thoracostomy tube was placed with drainage of 400 ml of blood. The patient was transported via air to our trauma center with an estimated transfer time of 30 min, during which he was transfused 8 units of packed red blood cells. Upon arrival, he had a Glascow Coma Scale of 3T, tachycardia at 160 bpm, and blood pressure was not detectable. Given the transmediastinal trajectory, the patient was not removed from the prehospital stretcher and a brief primary survey and roll was completed. A single wound was noted on his left chest, near the 3rd intercostal space left parasternal, and a cardiac focused assessment with sonography for trauma exam revealed a large pericardial effusion. Chest tube output was approximately 2000 ml. The patient was taken directly to the operating room by the trauma team.

**Fig. 1:**
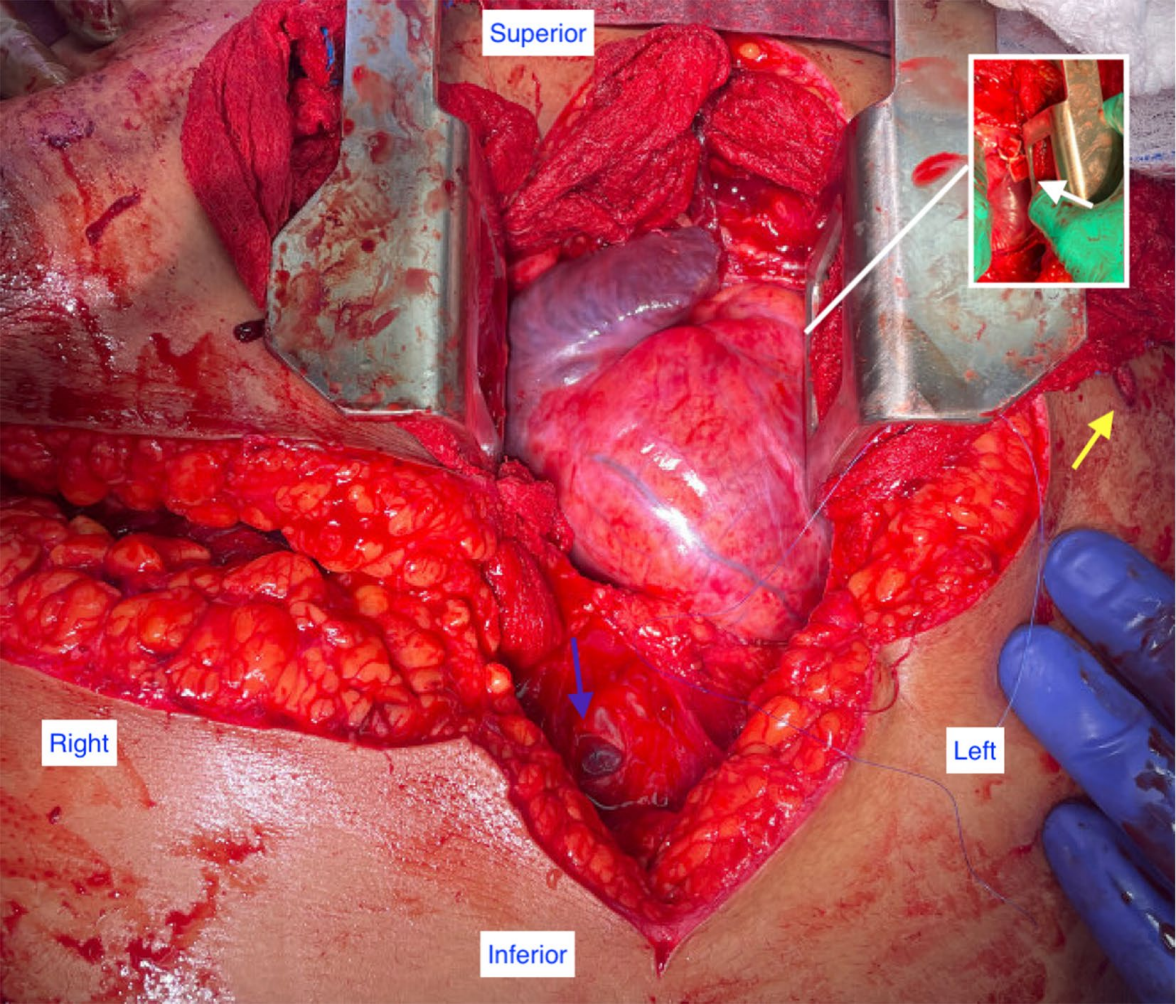
Intraoperative photo demonstrates the bullet entry site (yellow arrow), the RV injury after repair with pledgeted sutures (white arrow) and a 2 cm hole of the pericardium – exit site (blue arrow)

A median sternotomy was performed, the pericardium was opened and large amounts of blood clots were evacuated. A bleeding site near the inferior cavo-atrial junction was identified, which was controlled by occluding the hole with a finger. This allowed for time to volume resuscitate and stabilize the patient hemodynamically. The site was repaired primarily with pledgeted sutures. The patient suffered ventricular fibrillation cardiac arrest that responded to cardiac massage, volume resuscitation, and defibrillation. The remainder of the heart was inspected and a bleeding site in the right ventricular outflow tract, previously tamponaded by the sternum, was identified and controlled with skin staples and reinforced with pledgeted sutures. Further visual inspection of the heart revealed no other myocardial injuries, the coronaries arteries were distant from the bullet trajectory and appeared intact [Fig. 1].

The patient was now coagulopathic and had substantial ongoing bleeding from the right chest. Given the bullet trajectory and ongoing hemorrhage, a right thoracotomy was performed with evacuation of large amounts of blood. Bleeding sites were identified in the middle and lower lobes of the lung and were controlled with primary repair. Once bleeding was felt to be controlled and hemodynamics were acceptable, the patient was transferred with the chest packed open to the intensive care unit.

Transesophageal echocardiography was performed in the intensive care unit to identify injury to intracardiac structures. This revealed multiple mobile linear echodensities consistent with injury of the tricuspid valve apparatus including the papillary muscles and tricuspid chordae. Torrential tricuspid regurgitation and right ventricular dilatation with segmental akinetic areas of the outflow tract were identified. There was no evidence of intracardiac shunts. CXR and subsequently computed tomography (CT) of the chest revealed the retained bullet in the right lower chest wall below the scapula. Coagulopathy improved and the patient underwent chest washout and closure the following day.

CT scan of his head revealed a right frontal hypodensity and patient’s neurologic status was still unclear. He had persistent high grade fever early on and was placed on antibiotics. Given his significant volume overload from the massive blood and fluid resuscitation, the decision was made to delay tricuspid valve surgery for several days while recovering from these ongoing clinical issues.

Hemodynamic agents, which included epinephrine, milrinone, and epoprostenol, were gradually weaned. Patient had non focal neurologic exam and was following simple commands, so the decision was made to proceed with extubation on postoperative day three, with the thought that he was tolerating the severe tricuspid regurgitation and that repair could be performed on a more semi-elective basis. He was subsequently re-intubated due to increased work of breathing the following day and CT Scan of the chest revealed acute segmental and subsegmental pulmonary emboli; these were felt to be related to his acute trauma and massive blood transfusions. His lower extremities were negative for deep vein thrombosis. Due to fever and positive sputum cultures, he was also treated for ventilator associated pneumonia.

On postoperative day 5, he developed a mild elevation of his transaminases (AST 251 U/l, ALT 114 U/l) and later that day elevation of his creatinine (1.45 mg/dl) and lactate (12.3 mmol/l). Transthoracic echocardiography confirmed again severe TR, moderately dilated right ventricle (RV) and diastolic flattening of the septum consistent with RV volume overload. Placement of a pulmonary artery catheter was attempted but was not possible due to the degree of regurgitation. With signs of cardiogenic shock, acute renal and hepatic failure, and a rising lactate, decision was made for for peripheral VA-ECMO cannulation.

Due to body habitus (BMI 35, with predominant lower body obesity) and evidence of small femoral vessels on ultrasound, the patient was taken to the operating room for open surgical cannulation. ECMO was instituted via an open left common femoral artery approach (17 Fr. cannula), including an antegrade limb perfusion catheter to the left superficial femoral artery (7 Fr. sheath), and percutaneous right femoral venous drainage (23/25 Fr. cannula). Postoperatively, his hemodynamics improved, while his ECMO support achieved flow of 4.3 L per minute (LPM) with FiO2 of 100% and sweep gas flow at 3 LPM. Since he had been initiated on inotropic support, low dose milrinone and low dose epinephrine were maintained and weaned slowly over the next few days. His transaminases peaked on the day following initiation of ECMO (ALT 2218 U/l, AST above assay > 4000). His hemodynamic parameters along with lactate, creatinine and transaminases continued to improve, however, his bilirubin continued to rise. After ensuring proper positioning of the venous drainage cannula and ruling out any ultrasound detectable pathology of the liver, the decision was made to proceed with surgical correction of his tricuspid valve. On the 4th day of ECMO support, he underwent tricuspid valve surgery. The anterior leaflet of the tricuspid valve was destroyed including multiple chordae up to the level of the papillary muscle. Tricuspid valve replacement with a 29 mm porcine St Jude Epic bioprosthetic valve, epicardial lead implantation and exchange of his ECMO circuit was performed. An intraoperative weaning trial of his ECMO revealed a low pulmonary artery pulsatility index (PAPI) score, and the decision was made to remain on ECMO support. He was able to be weaned off his ECMO support 3 days following his valve surgery. His RV function had fully recovered. He failed extubation and ultimately required tracheostomy for respiratory failure. He remained in the hospital until his tracheal decannulation and was discharged to rehabilitation 55 days following his initial injury.

## Discussion and conclusions

Penetrating cardiac injuries are associated with high mortality rates ranging between 29% and 86% [3, 8, 9]. Estimates suggest under 10% of those with penetrating cardiac injuries reach the hospital alive [6]. Gunshot wounds have significantly higher pre-hospital and in-hospital mortality than stab wounds [3, 6, 10, 11]. Favorable prognostic characteristics have been reported to include the presence of tamponade, right ventricular injury, single chamber involvement, and the absence of pleural breach [3, 10–13]. 

In this report, the patient survived a two-chamber cardiac injury with violation of the pleural space. Despite the need for transfer from another facility, with the associated delay in definitive care, it allowed for the patient to be initiated on fluid and blood product resuscitation as well as placement of a thoracostomy tube prior to arrival to our facility. Pericardial tamponade is often reported to confer survival advantage, presumably by limiting acute blood loss at the expense of cardiac chamber compression, but this effect is likely limited and time dependent [11, 14]. We believe that the pattern of injury in this patient was favorable as the pericardial opening draining into the pleural space was small enough to prevent exsanguination, but sufficient to provide relief once intrapericardial volume and pressure increased. Without this pericardial “relief valve”, tamponade physiology would have likely progressed rapidly during resuscitative efforts.

The approach to access the transmediastinal injury was median sternotomy. When tamponade is suspected, sternotomy allows for a rapid relief of the tamponade but also provides easy access to all aspects of the heart over a thoracotomy approach. This is especially preferred when there is no suspicion of major extracardiac thoracic injuries [11]. 

The decision to delay definitive correction of his tricuspid valve injury was guided by basic principles of trauma, namely hemorrhage control, resuscitation and plan for re-exploration. The patient exhibited acceptable hemodynamics following bleeding control and resuscitation, therefore further cardiac surveillance with echocardiography was deferred to a later time while in the intensive care unit. Although the bullet trajectory suggested a possible valve or septal injury, we decided not to pursue any further investigation as this would have not provided any immediate survival benefit in the presence of severe coagulopathy and unknown neurologic function. Tricuspid regurgitation is usually well tolerated even in acute settings (such as in trauma or endocarditis) [15–17]. There has been one report of a patient requiring immediate correction of tricuspid regurgitation at the time of the surgical management of the injury, however this was a combined injury including an atrial septal defect; the latter having possibly caused excessive volume burden to the right heart leading to an acute decompensation [17]. In our case, an acute decompensation occurring five days after the injury was likely related to the patient’s worsening pulmonary status. As the right ventricle is highly sensitive to afterload, the patient’s hypoxia, need for positive pressure ventilation, and presence of small pulmonary emboli all appear to have contributed to excessive right heart afterload. This combined with the high intravascular volume following resuscitation likely led to RV volume overload, dysfunction and subsequent cardiogenic shock. Despite the known torrential tricuspid regurgitation, ECMO institution was not indicated earlier since there was no sign of RV decompensation and organ dysfunction related to this.

Management of trauma patients with extracorporeal circulation has been described in the literature. While in the acute setting, use of cardiopulmonary bypass may be necessary to address potential injuries, this is rather rare [3]. The use of ECMO has an advantage in that it does not require high anticoagulation goals and cannula sizes are usually smaller. It is more often utilized in the setting of blunt trauma to support patients in acute respiratory distress syndrome, and is uncommonly utilized for myocardial contusion or cardiogenic shock due to intracardiac injuries. Despite the ability to flow with no anticoagulation, the presence of pulmonary emboli favored institution of anticoagulation. Our case highlights the novel use of ECMO for cardiogenic shock secondary to traumatic tricuspid valve injury. Similar to other indications for mechanical circulatory support in cardiogenic shock, ECMO was favored in this case over a single heart assist device (such as right Impella or Protek Duo) due to severe and rapid metabolic derangements, and because of the ability to decrease the volume burden and subsequent congestive hepatopathy.

Tricuspid valve repair is accepted as superior to replacement, especially in younger patients. The degree of tissue damage in this patient, would have required a very complex repair with protracted cardiopulmonary bypass with a questionable short or long term result in this patient whose hepatic injury had not fully recovered. Interventional/catheter based approaches are often used as “off label procedures” in functional tricuspid regurgitation; repair of the detached anterior leaflet with acceptable degree of residual regurgitation would have not been possible. Replacement assured a fast intervention and the best chance to ameliorate his congestive hepatopathy and shock liver by allowing for no residual tricuspid regurgitation.

This is the first report, to our knowledge, in which ECMO was utilized in the management of penetrating traumatic tricuspid valve injury prior to definitive management with valve replacement surgery, and highlights ECMO as an invaluable resource in penetrating cardiac injuries. We suggest that in cases where isolated cardiac injury is the main contributor to multi-system organ failure, ECMO should be considered for management of these trauma patients.

## Data Availability

All data and materials analysed in the current report are not publicly available due to patient privacy but are available from the corresponding author upon reasonable request.

## Abbreviations

ALT: Alanine transaminase
AST: aspartate transaminase
CT: Computed tomography
ECMO: extracorporeal-membrane oxygenation
GSW: gunshot wound
RV: right ventricle
TR: tricuspid regurgitation
VA: veno-arterial

## References

[1] Goldstick JE, Carter PM, Cunningham RM (2021). Current epidemiological trends in Firearm Mortality in the United States. JAMA Psychiatry.

[2] Goldstick JE, Zeoli A, Mair C, Cunningham RM (2019). US Firearm-related mortality: National, State, and Population trends, 1999–2017. Health Aff (Millwood).

[3] Morse BC, Mina MJ, Carr JS (2016). Penetrating cardiac injuries: a 36-year perspective at an urban, Level I trauma center. J Trauma Acute Care Surg.

[4] Cunningham RM, Walton MA, Carter PM (2018). The major causes of death in children and adolescents in the United States. N Engl J Med.

[5] Kang N, Hsee L, Rizoli S, Alison P (2009). Penetrating cardiac injury: overcoming the limits set by Nature. Injury.

[6] Campbell NC, Thomson SR, Muckart DJ, Meumann CM, Van Middelkoop I, Botha JB (1997). Review of 1198 cases of penetrating cardiac trauma. Br J Surg.

[7] Wang C, Zhang L, Qin T et al. Extracorporeal membrane oxygenation in trauma patients: a systematic review. World J Emerg Surg. 2020;15(1):51. Published 2020 Sep 11. 10.1186/s13017-020-00331-2. 10.1186/s13017-020-00331-2 PMC7488245 32912280

[8] O’Connor J, Ditillo M, Scalea T (2009). Penetrating cardiac injury. J R Army Med Corps.

[9] Mandal AK, Sanusi M (2001). Penetrating chest wounds: 24 years experience. World J Surg.

[10] Tang AL, Inaba K, Branco BC (2011). Postdischarge complications after penetrating cardiac injury: a survivable injury with a high postdischarge complication rate. Arch Surg.

[11] Degiannis E, Loogna P, Doll D, Bonanno F, Bowley DM, Smith MD (2006). Penetrating cardiac injuries: recent experience in South Africa. World J Surg.

[12] Mina MJ, Jhunjhunwala R, Gelbard RB (2017). Factors affecting mortality after penetrating cardiac injuries: 10-year experience at urban level I trauma center. Am J Surg.

[13] Tyburski JG, Astra L, Wilson RF, Dente C, Steffes C (2000). Factors affecting prognosis with penetrating wounds of the heart. J Trauma.

[14] Moreno C, Moore EE, Majure JA, Hopeman AR (1986). Pericardial tamponade: a critical determinant for survival following penetrating cardiac wounds. J Trauma.

[15] Zhang Z, Yin K, Dong L (2017). Surgical management of traumatic tricuspid insufficiency. J Card Surg.

[16] Carozza A, Renzulli A, De Feo M (2001). Tricuspid repair for infective endocarditis: clinical and echocardiographic results. Tex Heart Inst J.

[17] Enomoto Y, Sudo Y, Sueta T (2015). Traumatic tricuspid insufficiency requiring valve repair in an Acute setting. Hellenic J Cardiol.

